# Supplementary material for: Arctic amplification is caused by sea-ice loss under increasing CO2
Source: Nat Commun. 2019 Jan 10;10:121. doi: 10.1038/s41467-018-07954-9 (PMC6328634; doi:10.1038/s41467-018-07954-9)
Supplement: Supplementary file 1 — Supplementary Information [file 41467_2018_7954_MOESM1_ESM.pdf]

Supplementary Information for

Arctic amplification is caused by sea-ice loss under increasing CO_2

Aiguo Dai, Dehai Luo, Mirong Song, and Jiping Liu

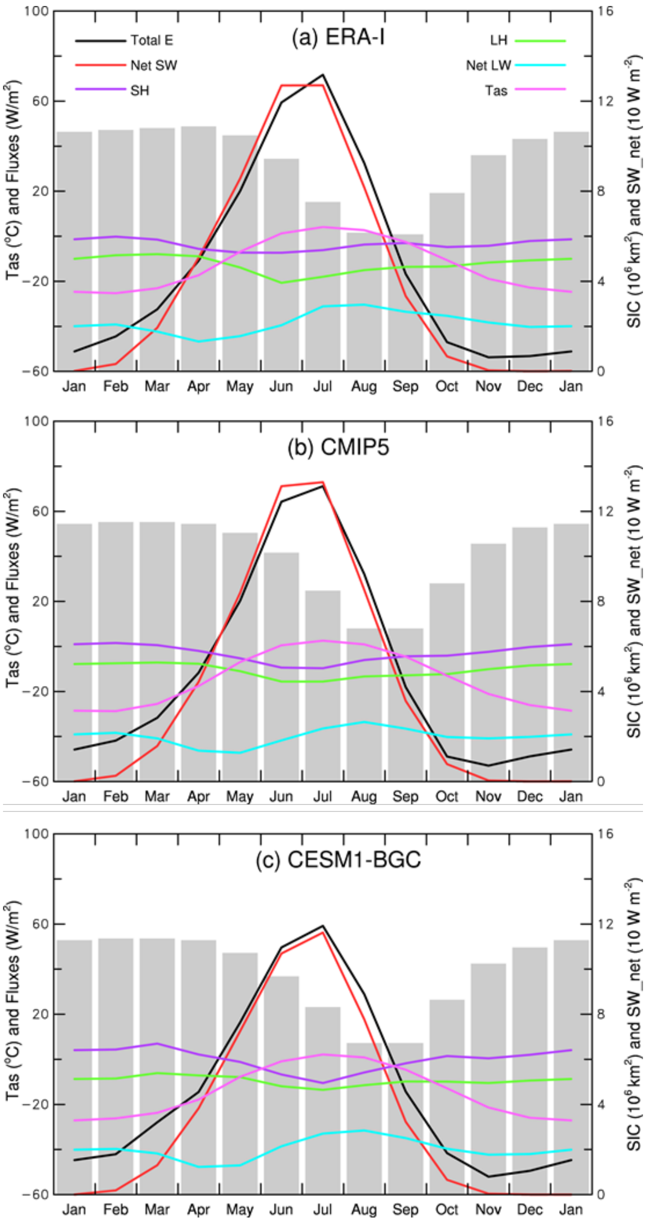

Figure 1. **1979-1988 mean annual cycle** of sea-ice cover (SIC, grey bars, right y-axis, in 10^6 km^2), surface air temperature (Tas), and surface energy fluxes averaged over the Arctic (67°-90°N) from (**a**) EAR-I, (**b**) the ensemble mean of historical simulations by 38 CMIP5 models, and (**c**) CESM1-BGC historical simulation. All fluxes are positive downward. SW=shortwave radiation, LW=longwave radiation, SH=sensible heat, LH=latent heat, black line =all fluxes combined. The CESM1-BGC is very similar to the CESM1 version used in our 1%CO2 runs.

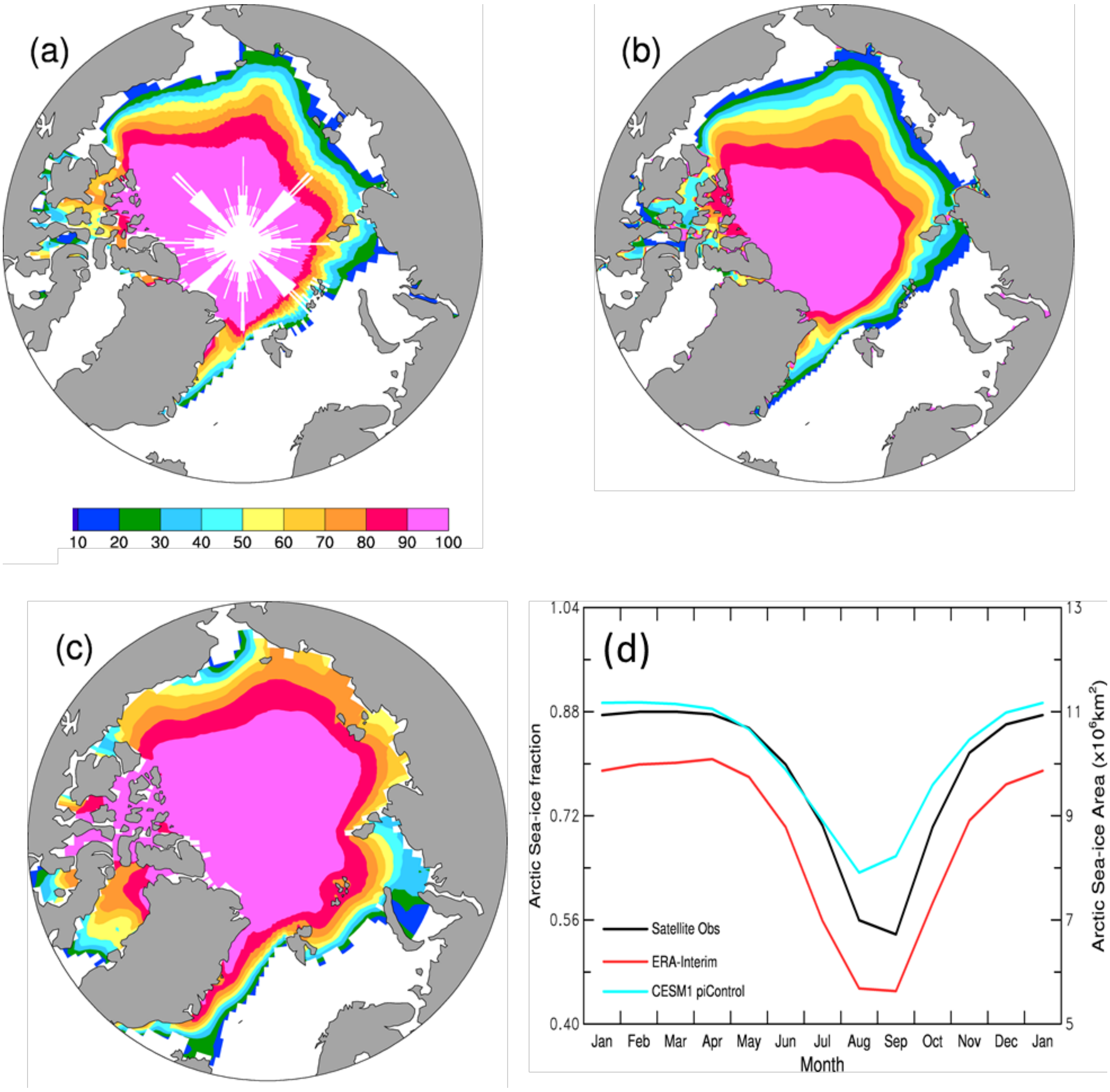

Figure 2. (**a**) 1979-2015 mean September SIC (%) from satellite observations (obtained from ftp://sidads.colorado.edu/DATASETS/NOAA/G02202_V3/). (**b**) Same as (**a**) but from ERA-Interim. (**c**) 80-year climatology of September SIC from the CESM1 pre-industrial control run. (**d**) The mean seasonal variations of the Arctic (67°-90°N) mean sea-ice fraction (left y-axis) and area cover (right y-axis). The higher SIC during summer months in the CESM1 piControl run is expected because of its cooler climate under pre-industrial CO2 level of 284.7ppmv.

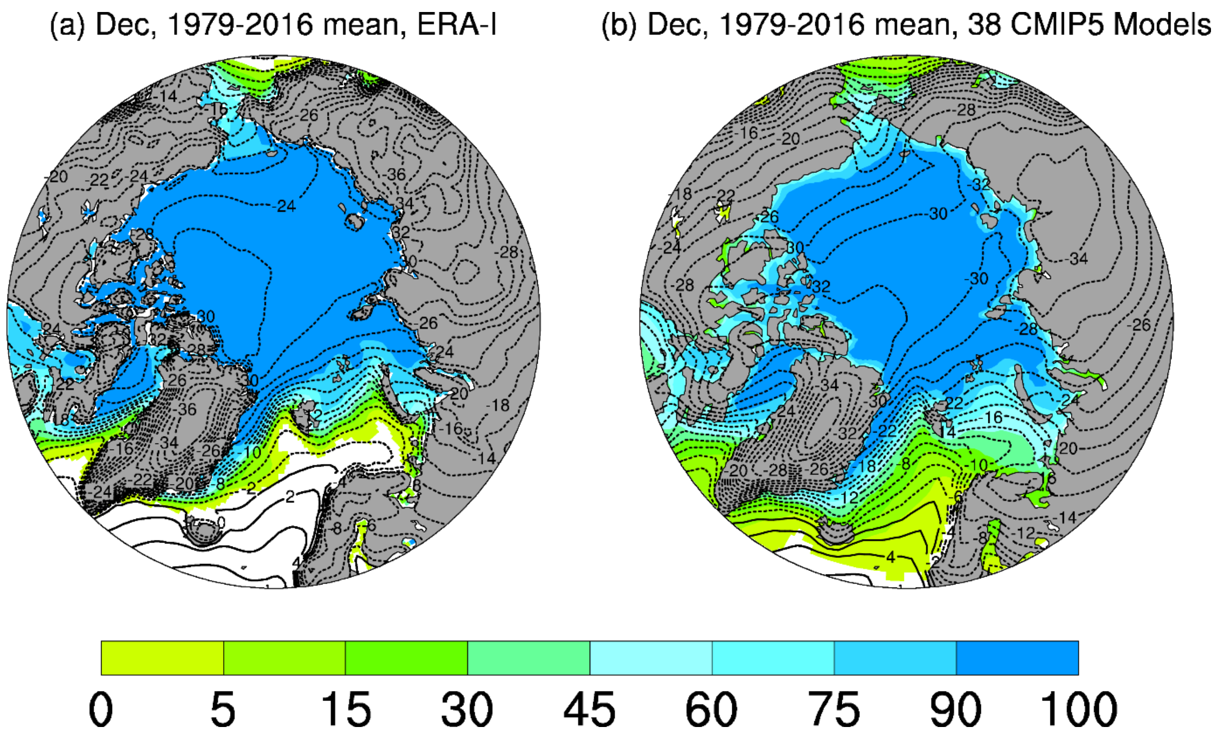

Figure 3. Distribution of 1979-2016 mean December sea-ice concentration (SIC, color shading, in %) and surface air temperature (contours, interval=2, in °C) from (**a**) ERA-Interim reanalysis data and (**b**) the ensemble mean of 38 CMIP5 models. Note the large temperature difference between the areas with and without sea-ice over the ocean.

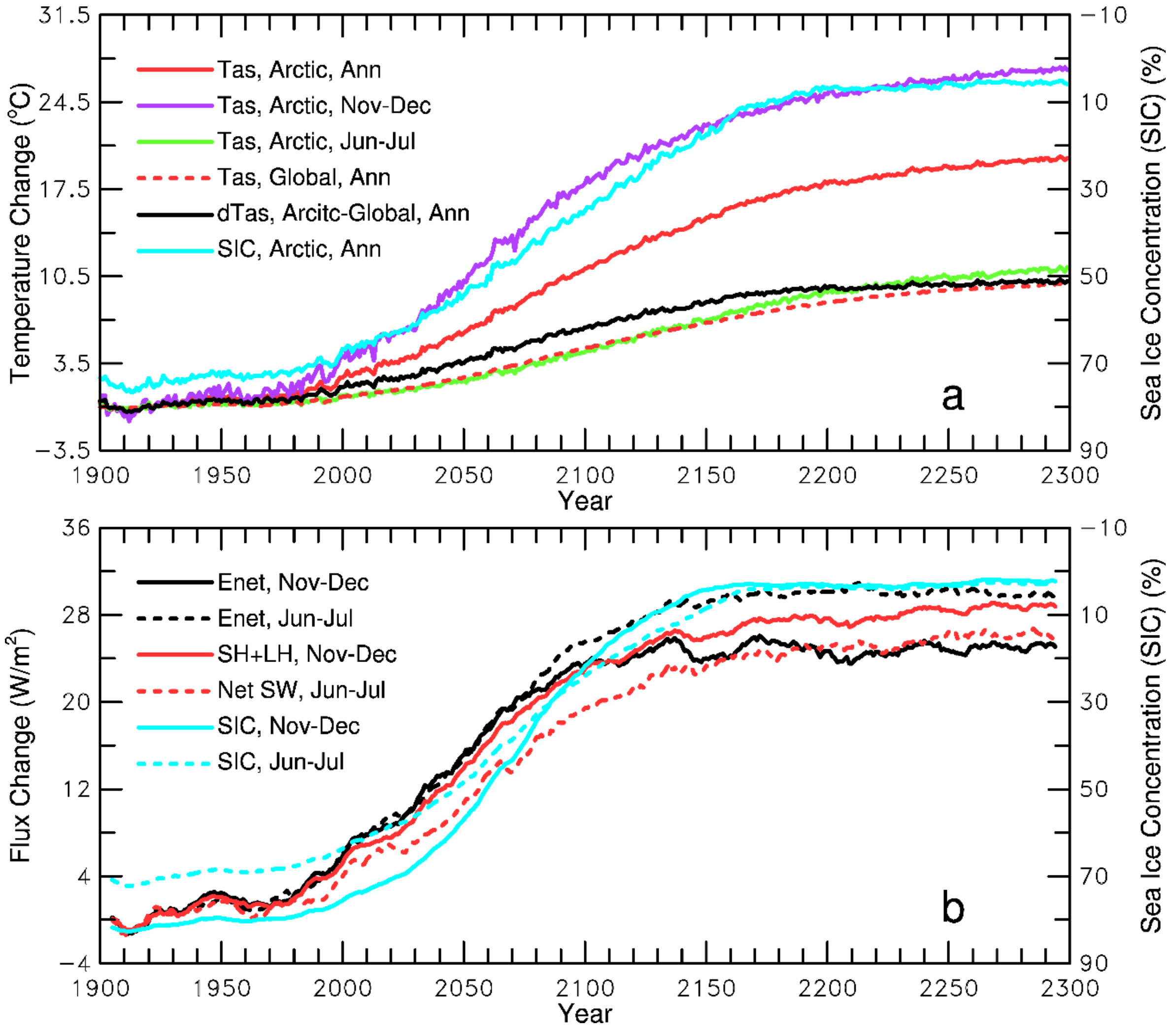

Figure 4. (a) Time series of Arctic (67°-90°N) and global mean surface air temperature (Tas) anomalies (relative to 1900-1929 mean), the annual Arctic Amplification (AA, black), and Arctic sea ice concentration (SIC, blue for annual, pink for Nov-Dec). Arctic Tas is shown for annual (red solid), Nov-Dec (magenta) and June-July (green). (b) Same as (a) but for Nov-Dec (solid) and June-July (dashed) mean Arctic surface net energy flux (Enet, black, positive upward for Nov-Dec but positive downward for June-July), Nov-Dec sensible plus latent heat flux (red solid, positive upward), June-July net shortwave flux (red dashed, positive downward), Nov-Dec (blue solid) and June-July (blue dashed) SIC. Time series in (b) were smoothed with 11-yr moving averaging. Nine CMIP5 models with data up to 2300 under the RCP85 scenario were included in this multi-model ensemble mean.

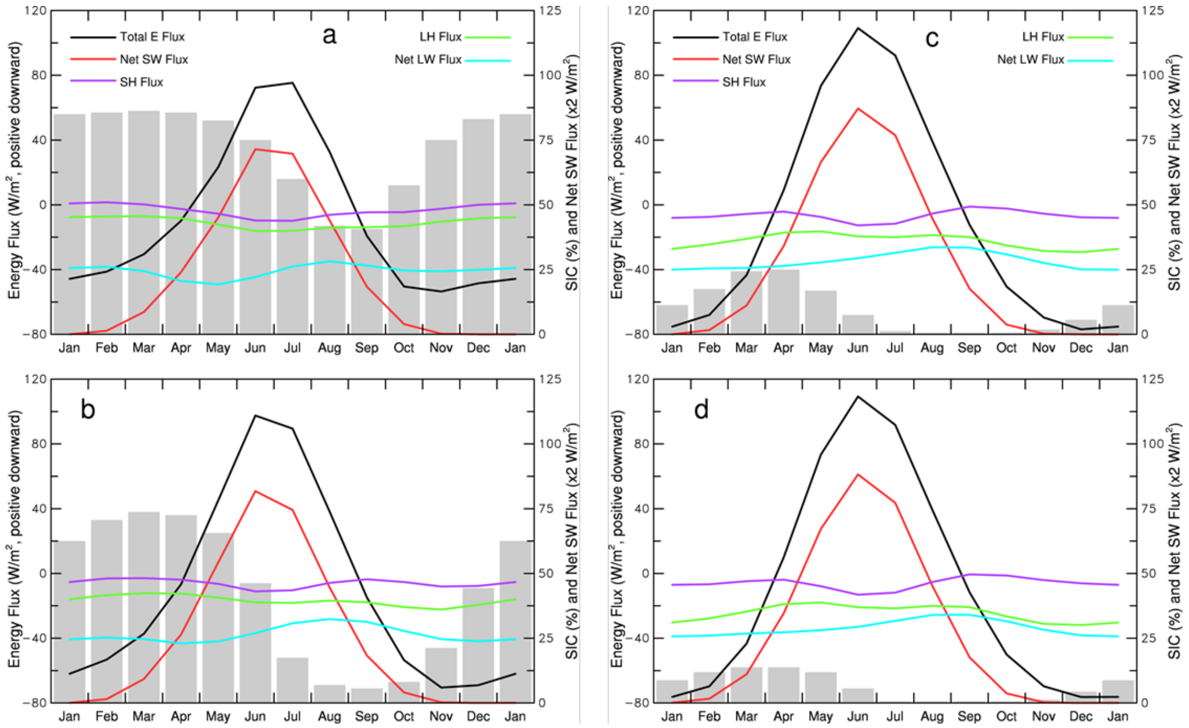

Figure 5. The mean annual cycle of Arctic (67°-90°N) SIC (shading, right ordinate) and surface energy fluxes (lines, positive downward, SW=shortwave radiation, LW=longwave radiation, SH=sensible heat, LH=latent heat, black line =all fluxes combined) for (a) 1970-1999, (b) 2070-2099, (c) 2170-2199, and (d) 2270-2299 under the historical and RCP85 scenarios from the ensemble mean of 9 runs from 9 CMIP5 models

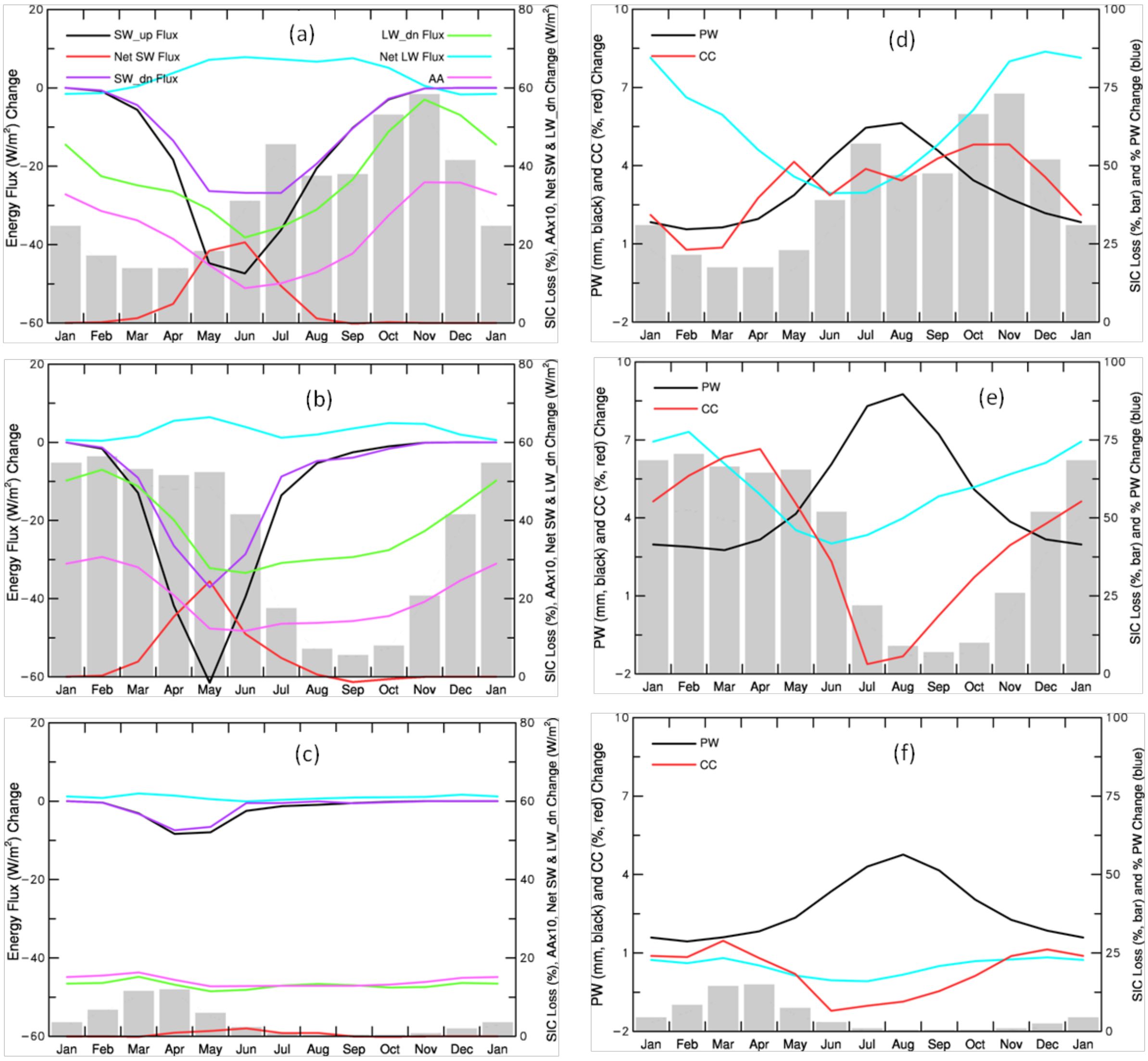

Figure 6. Left column: Centennial changes as a function of month in Arctic (67°-90°N) SIC (shading, right ordinate), the ratio of Arctic versus global warming (AA, right ordinate, multiplied by 10), and surface energy fluxes (lines, positive downward for net fluxes, on left ordinate except for net SW and downward LW; SW=shortwave radiation, LW=longwave radiation) for (**a**) 2070-2099 minus 1970-1999, (**b**) 2170-2199 minus 2070-2099, and (**c**) 2270-2299 minus 2170-2199 under the historical and RCP85 scenarios from the ensemble mean of 9 runs from the 9 CMIP5 models. **Right panels (d-f)**: Same as the right panels except for precipitable water (PW) and total cloud cover (CC). The blue line is the percentage PW change (on right ordinate).

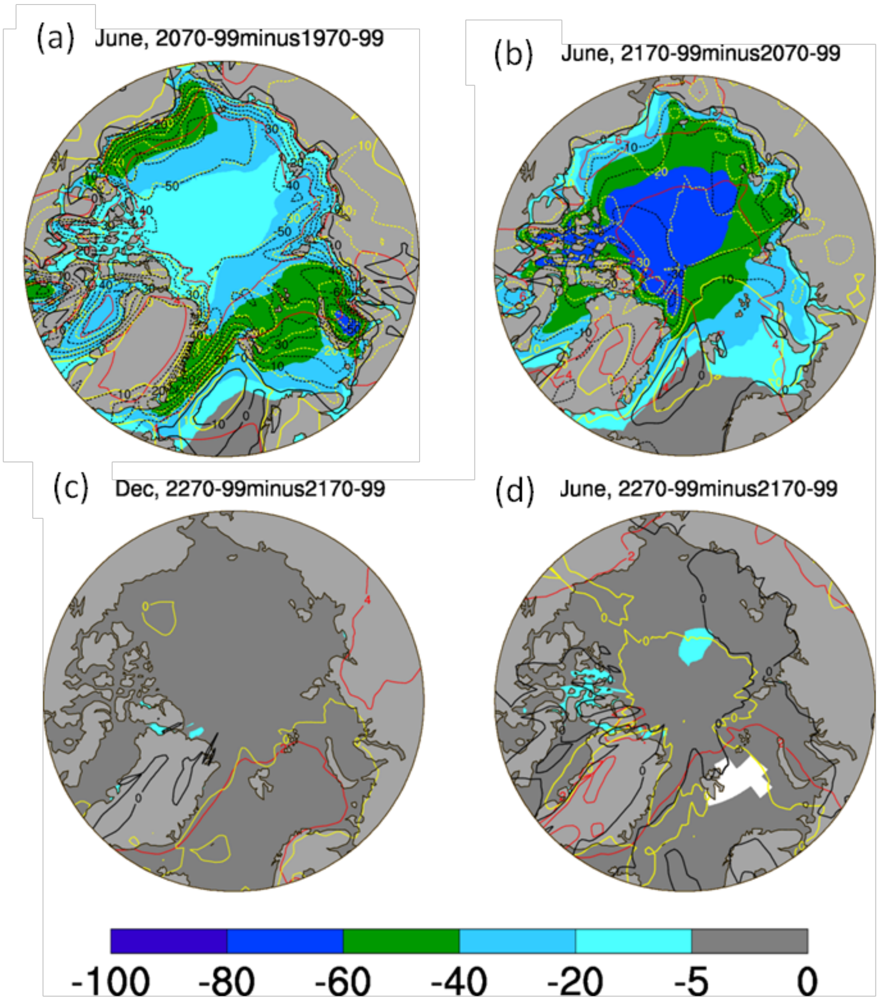

Figure 7. Centennial changes in June (a,b,d) and December (c) SIC (%, color shading), surface air temperature (^{o}C, red contours, interval=2), net surface energy flux (W/m^2, black contours, interval=10) and (yellow contours in W/m^2) surface latent and sensible heat flux in (c) or net surface shortwave radiation in (a, b, d). Dashed contours are for negative values. Fluxes are positive upward in this figure. Based on the ensemble mean of 9 simulations from 9 CMIP5 models under the historical and RCP85 scenarios.

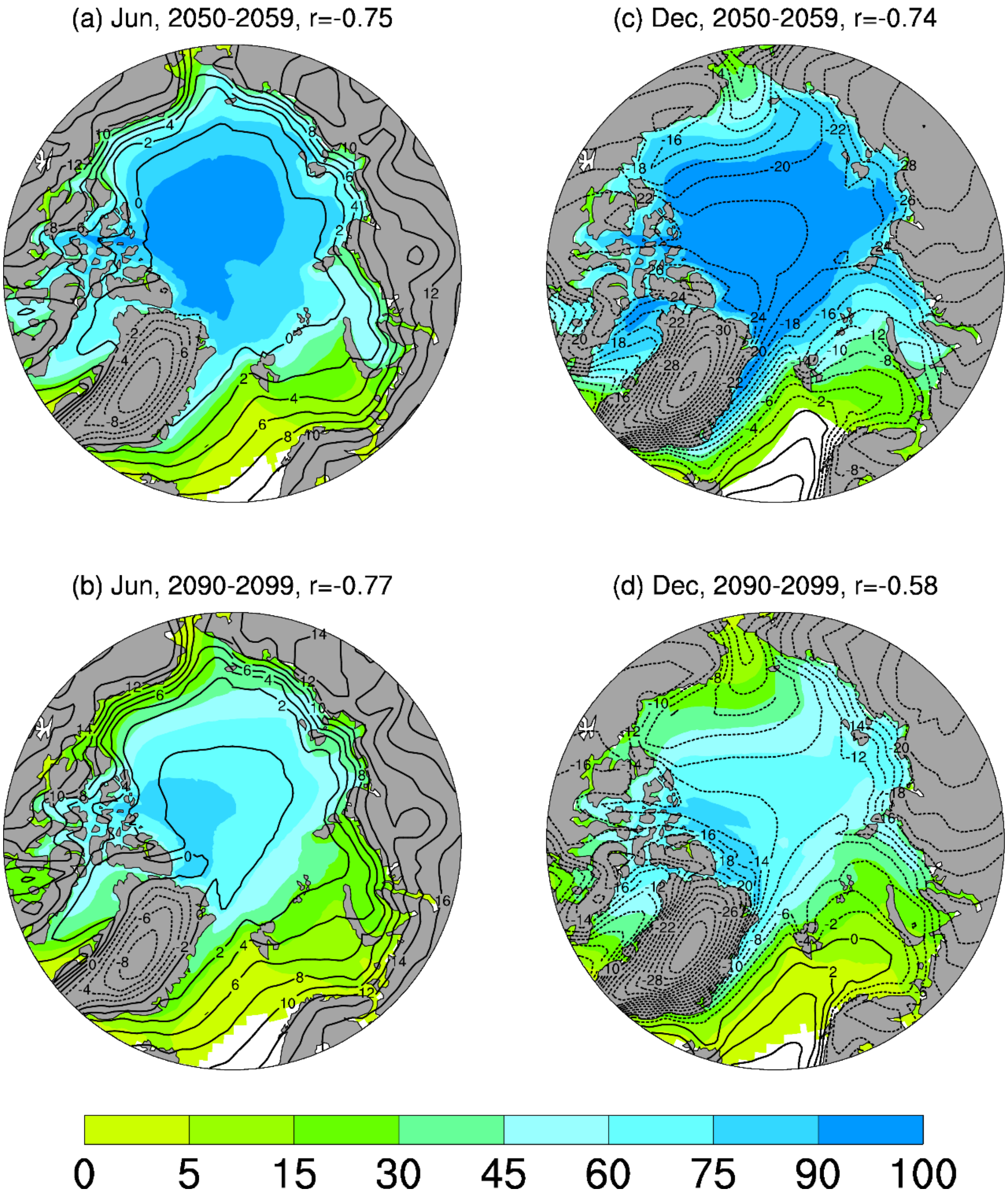

Figure 8. Spatial distributions of surface air temperature (contours, °C, interval=2, negative values in dashed lines) and sea-ice concentration (colors, %) for June (left) and December (right) during the 2050s (top) and 2090s (bottom). The spatial pattern correlation coefficient is given as *r* ($p<0.01$ for all cases). Derived from the ensemble mean of 38 simulations by 38 CMIP5 models under the RCP8.5 scenario.

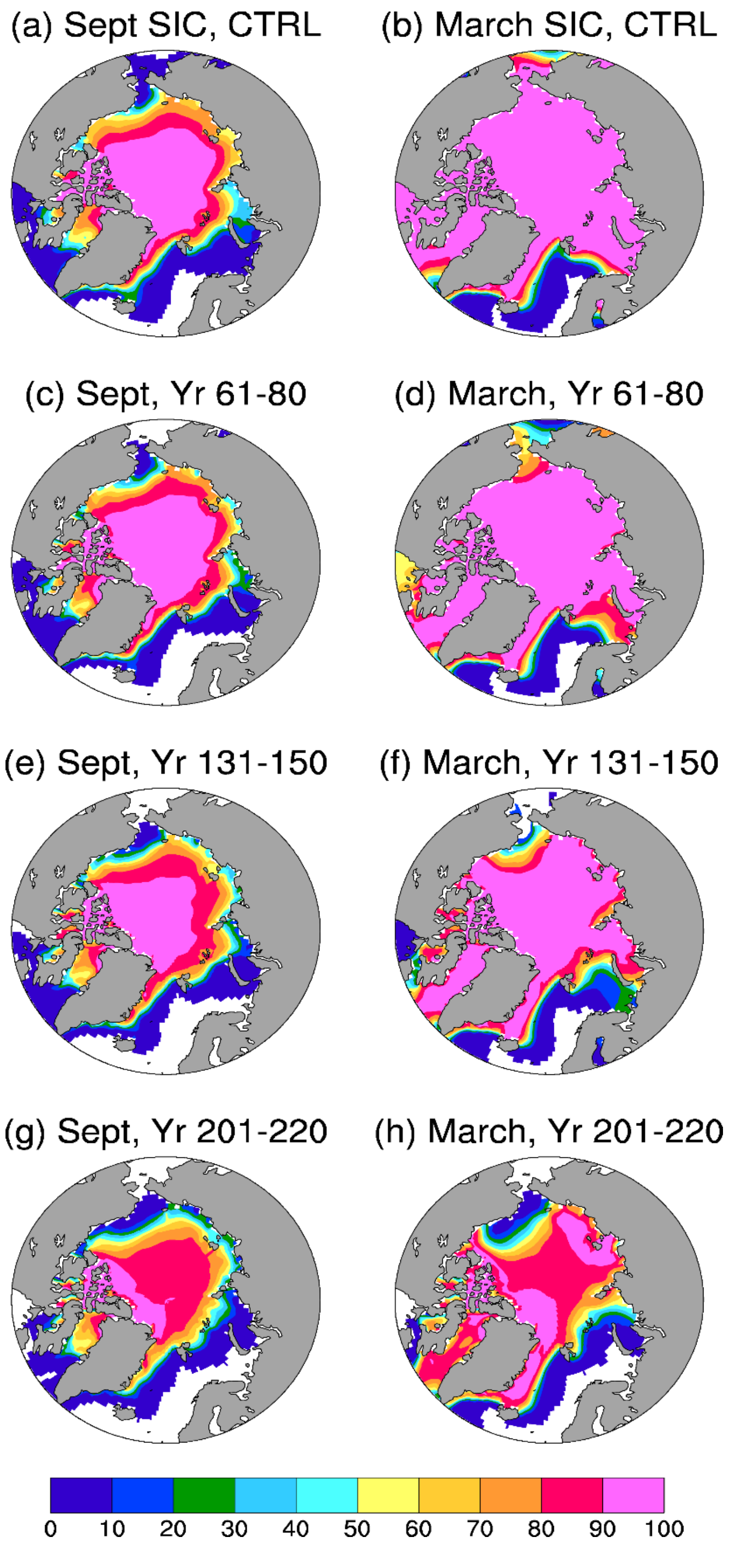

Figure 9. Mean Arctic sea-ice concentration (SIC, %) for September (left) and March (right) from the control run (top row) and the internally-calculated SIC from the FixedIce run averaged for the 20 year period around the 1st (2nd row), 2nd (3rd row) and 3rd (4th row) doubling of atmospheric CO_2. All colored areas have positive SIC where internally-calculated surface fluxes were used in the FixedIce run. Surface fluxes from the control run were used only over the lower latitude sea-ice margins where the initial sea-ice were melted away completely (i.e., only over the CTRL SIC fraction of the areas with color in the top row but in white in the lower rows).

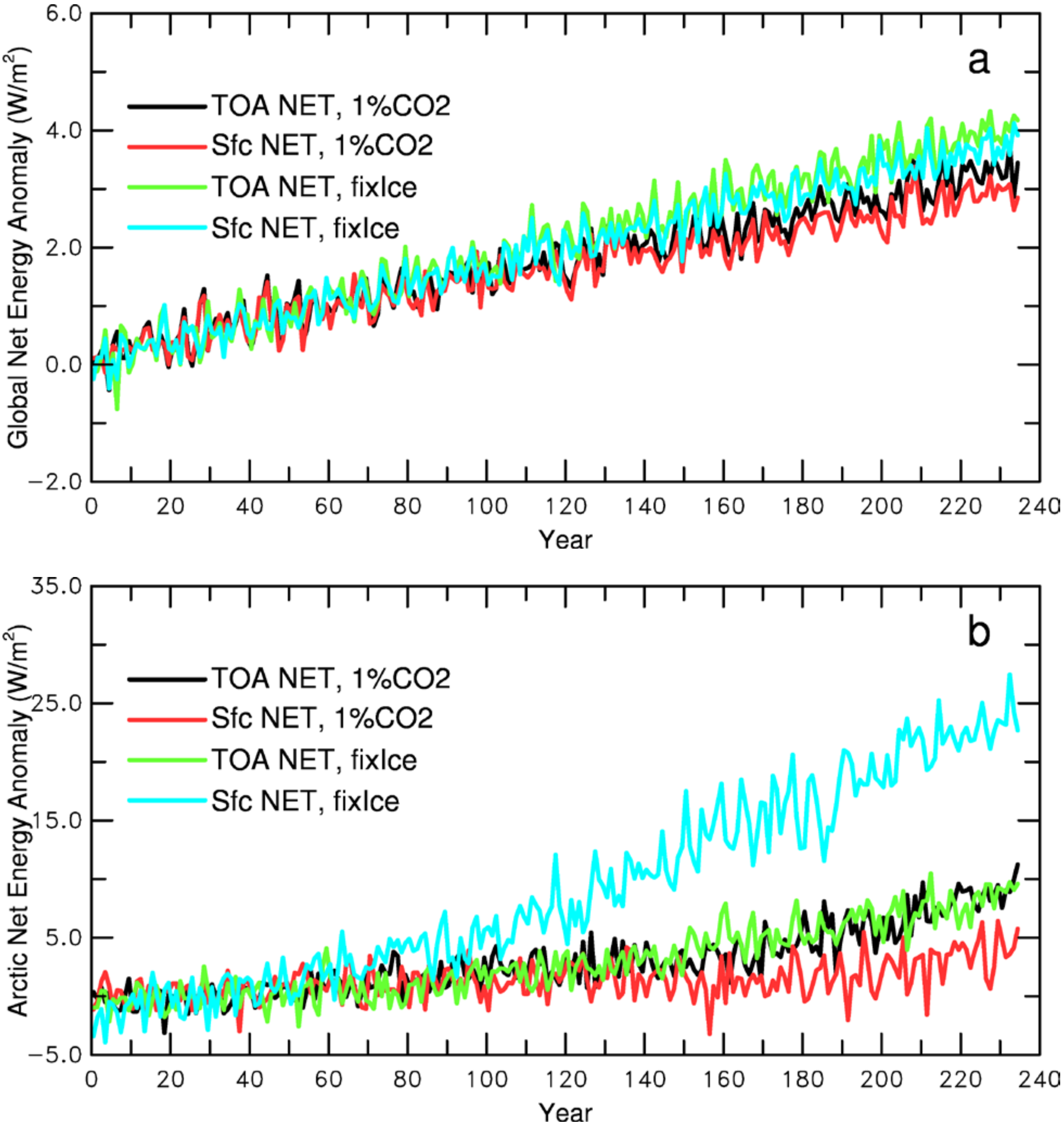

Figure 10. Changes (relative to control climatology) in annual (a) global-mean and (b) Arctic-mean net energy fluxes (positive downward) at the top-of-atmosphere (TOA) and surface from the standard 1%CO2 and fixed_Ice runs.

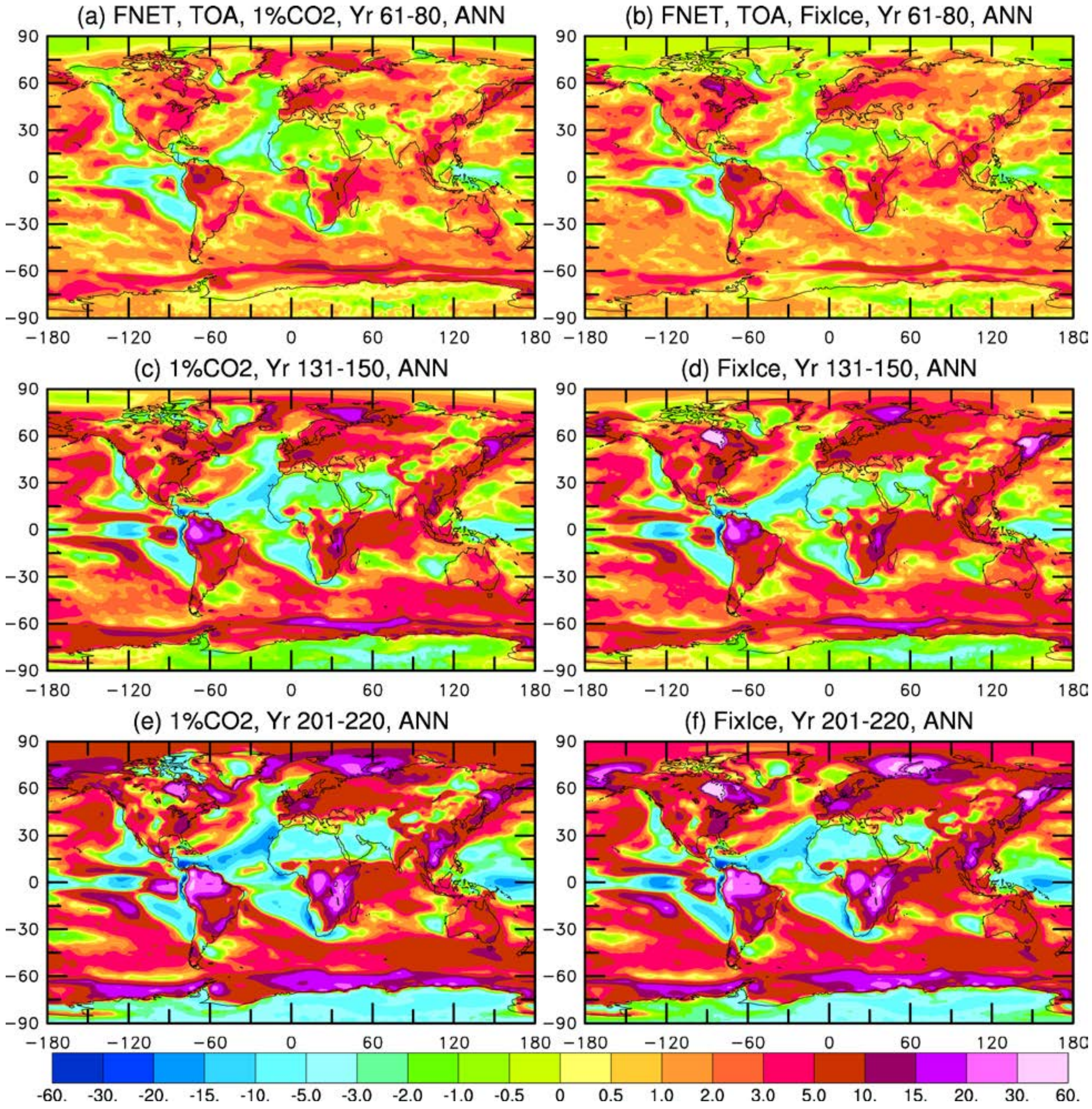

Figure 11. Changes (relative to control climatology) in top-of-atmosphere (TOA) annual net energy flux (W/m^2, positive downward) around the 1st (top row), 2nd (middle row) and 3rd (bottom row) doubling of atmospheric CO2 from the standard 1%CO2 (left) and Fixed_Ice (right) runs.

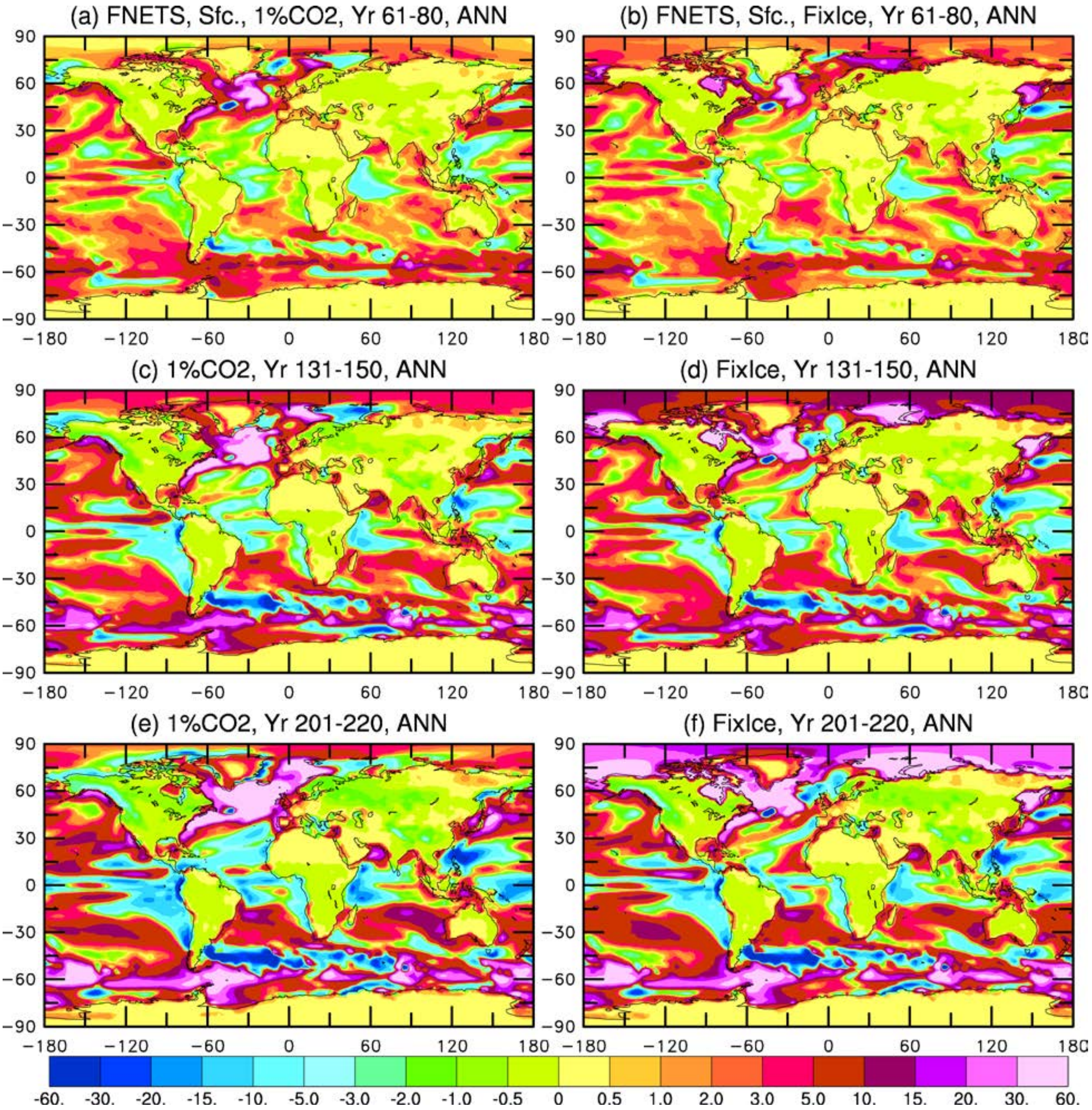

Figure 12. Changes (relative to control climatology) in annual surface net energy flux (W/m^2, positive downward) around the 1st (top row), 2nd (middle row) and 3rd (bottom row) doubling of atmospheric CO2 from the standard 1%CO2 (left) and Fixed_Ice (right) runs.

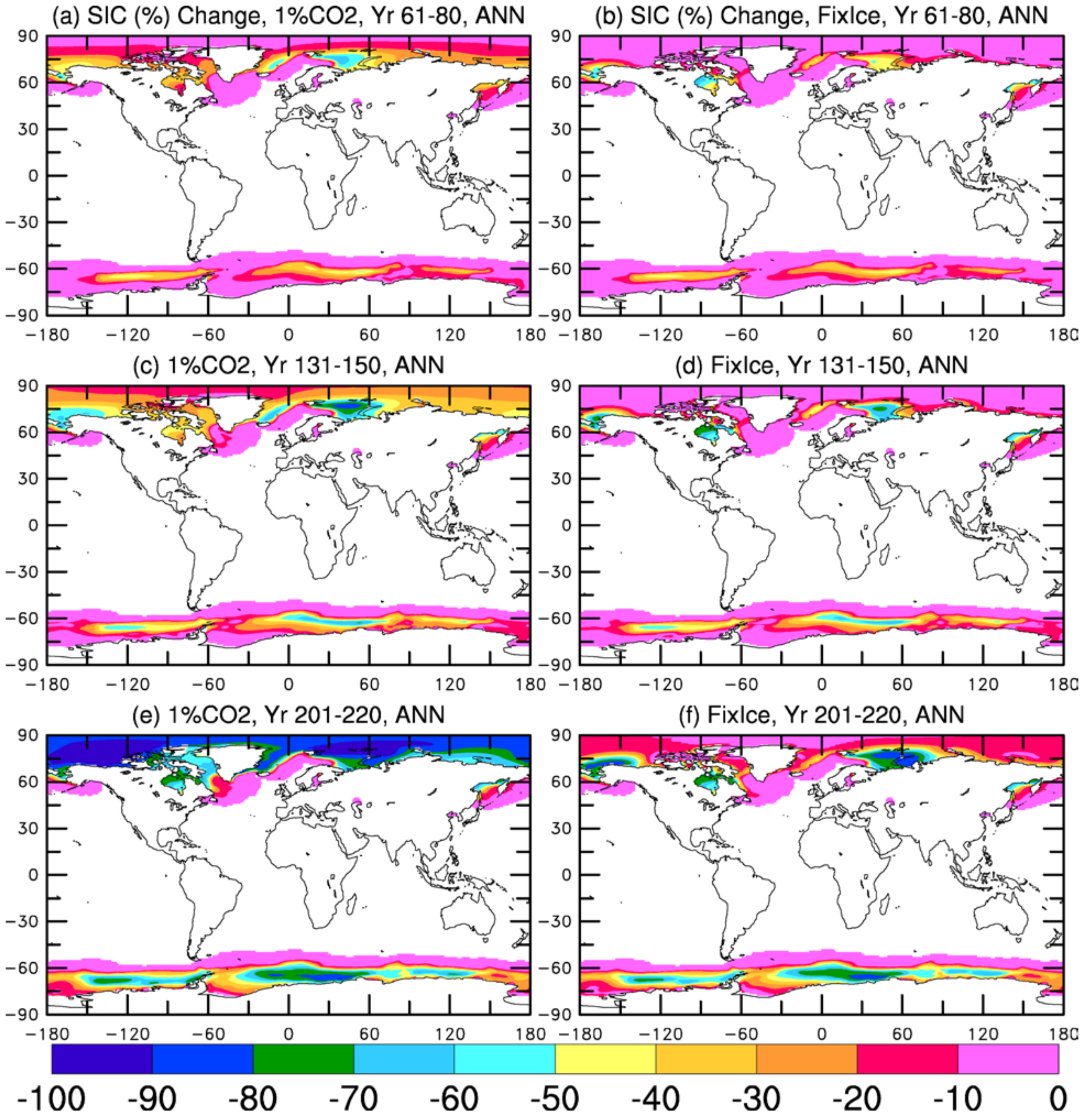

Figure 13. Changes (relative to control climatology) in annual sea-ice concentration (SIC, in % of grid area) around the 1st (top row), 2nd (middle row) and 3rd (bottom row) doubling of atmospheric CO2 from the standard 1%CO2 (left) and Fixed_Ice (right) runs.

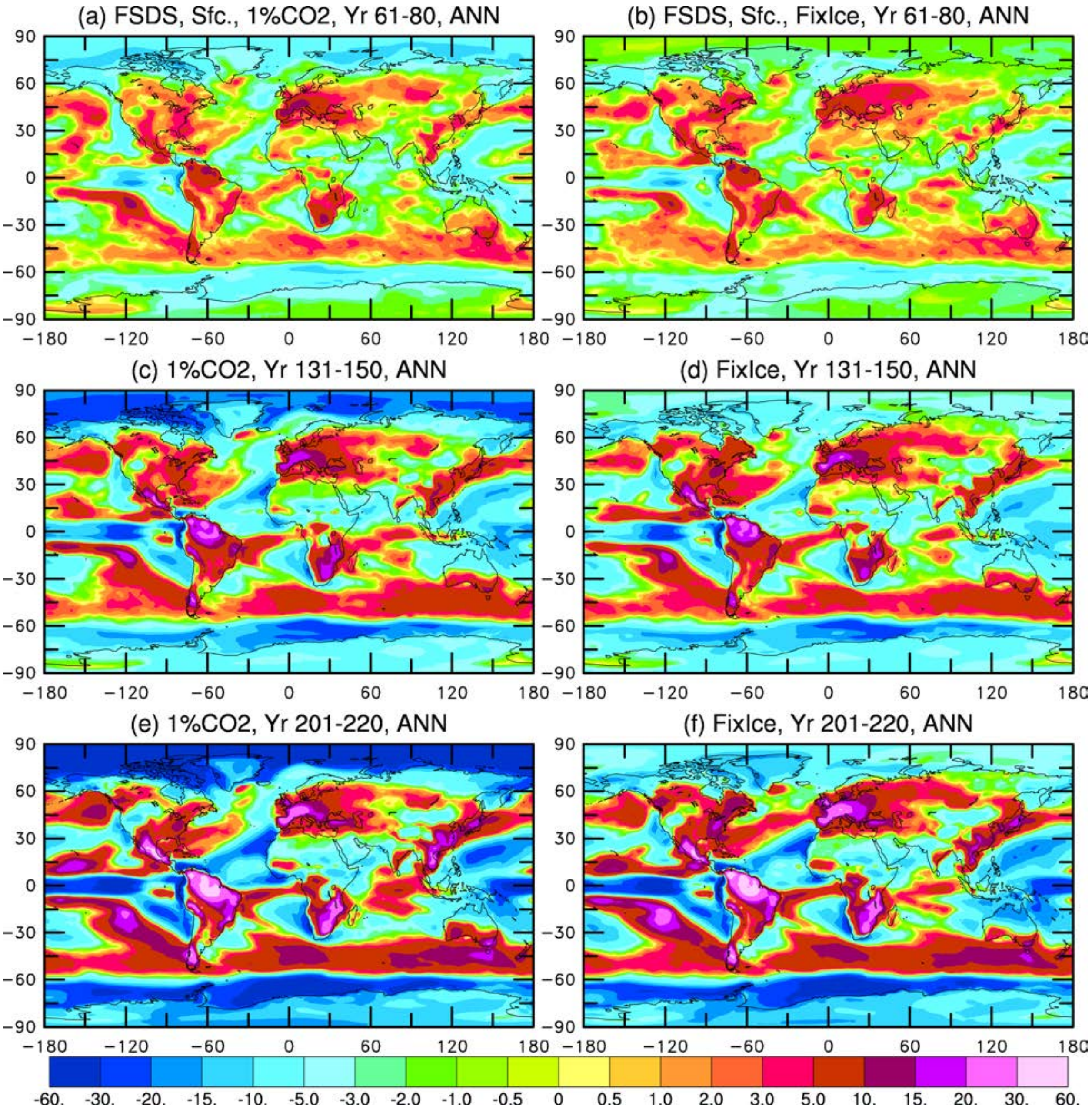

Figure 14. Changes (relative to control climatology) in annual surface downward shortwave radiative flux (W/m^2, positive downward) around the 1st (top row), 2nd (middle row) and 3rd (bottom row) doubling of atmospheric CO2 from the standard 1%CO2 (left) and Fixed_Ice (right) runs.

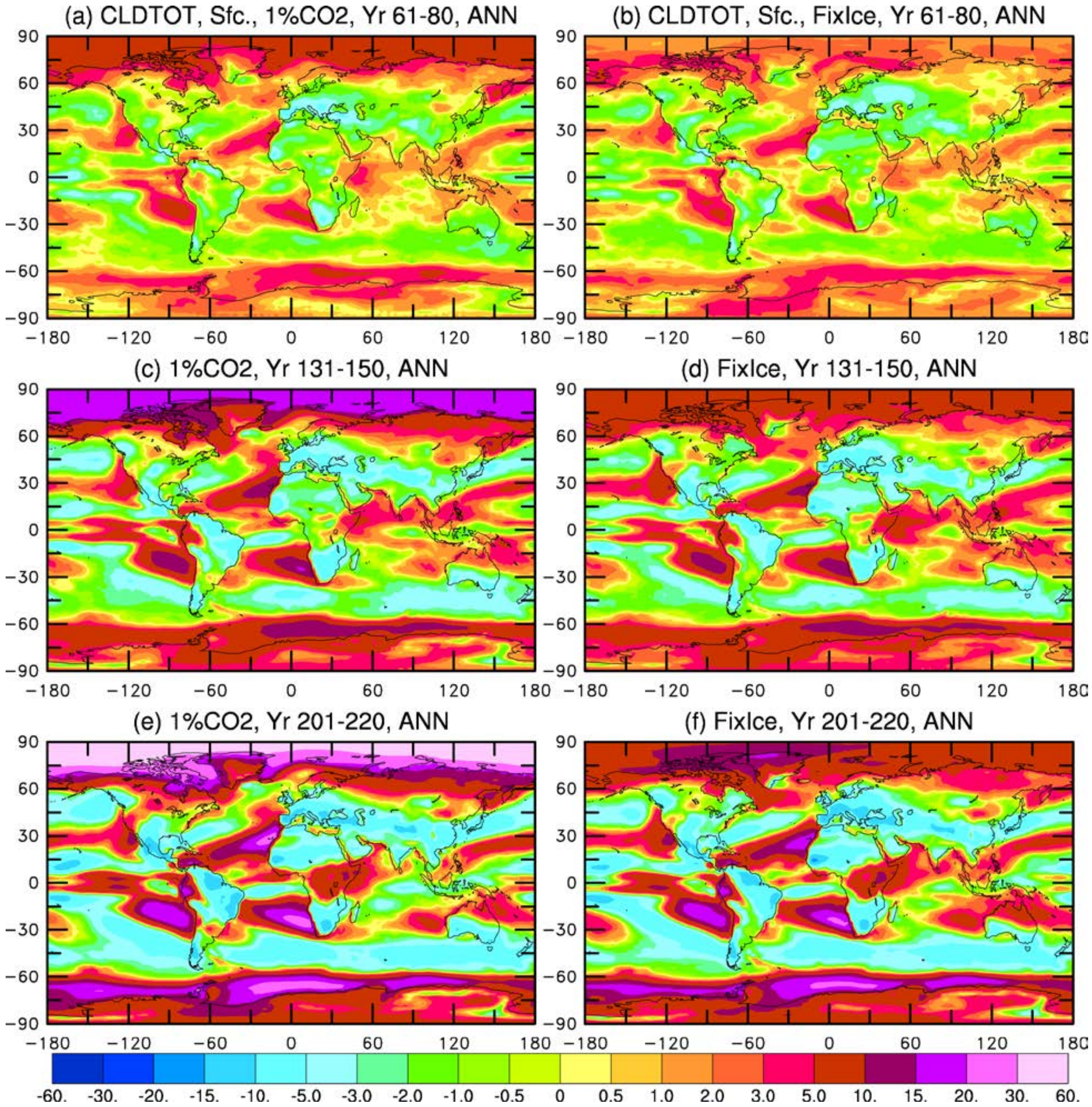

Figure 15. Changes (relative to control climatology) in annual total cloud cover (%) around the 1st (top row), 2nd (middle row) and 3rd (bottom row) doubling of atmospheric CO2 from the standard 1%CO2 (left) and Fixed_Ice (right) runs. The increase in Arctic clouds is seen in low, middle and high clouds.

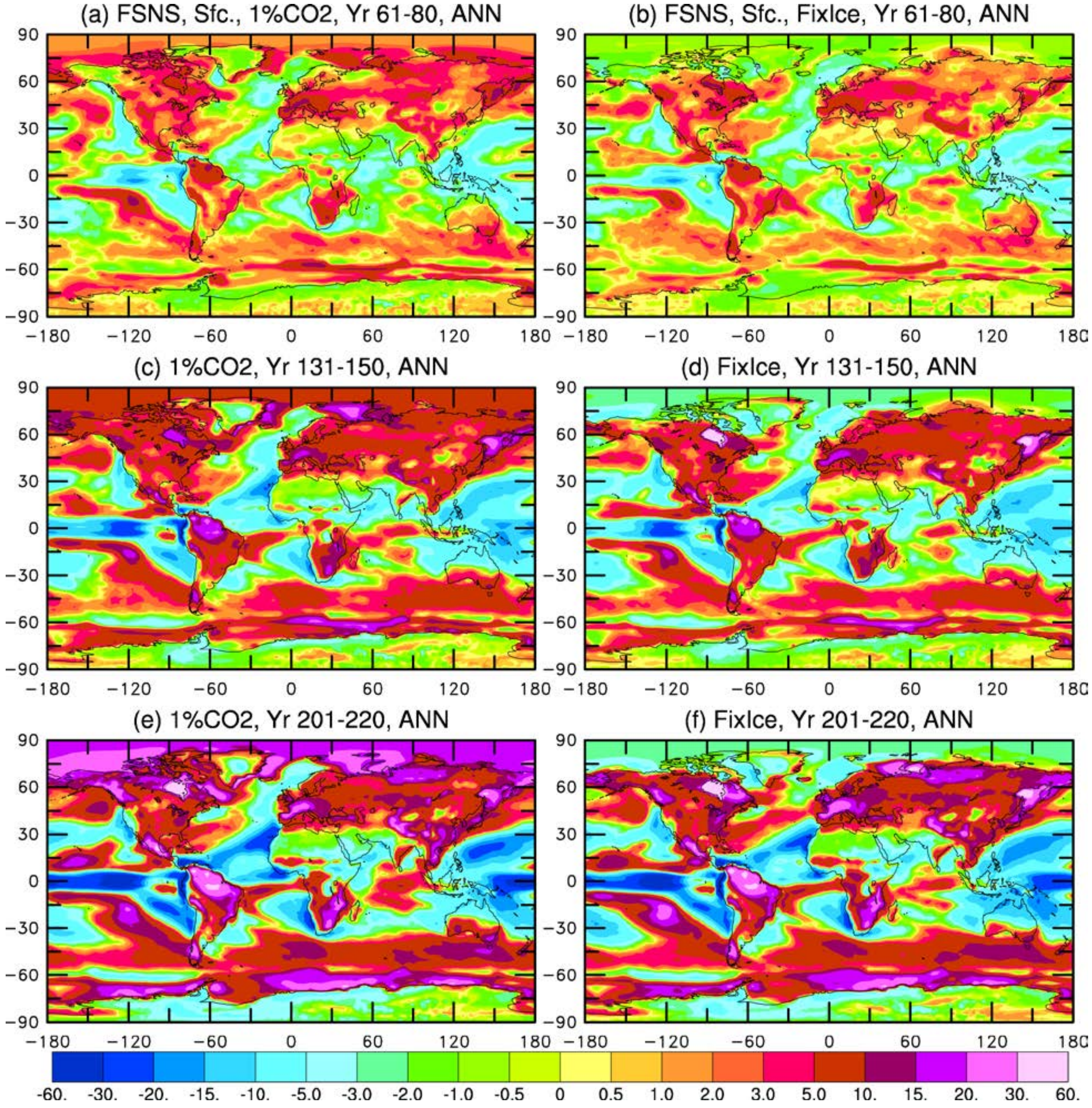

Figure 16. Changes (relative to control climatology) in annual surface net shortwave radiative flux (W/m^2, positive downward) around the 1st (top row), 2nd (middle row) and 3rd (bottom row) doubling of atmospheric CO2 from the standard 1%CO2 (left) and Fixed_Ice (right) runs.

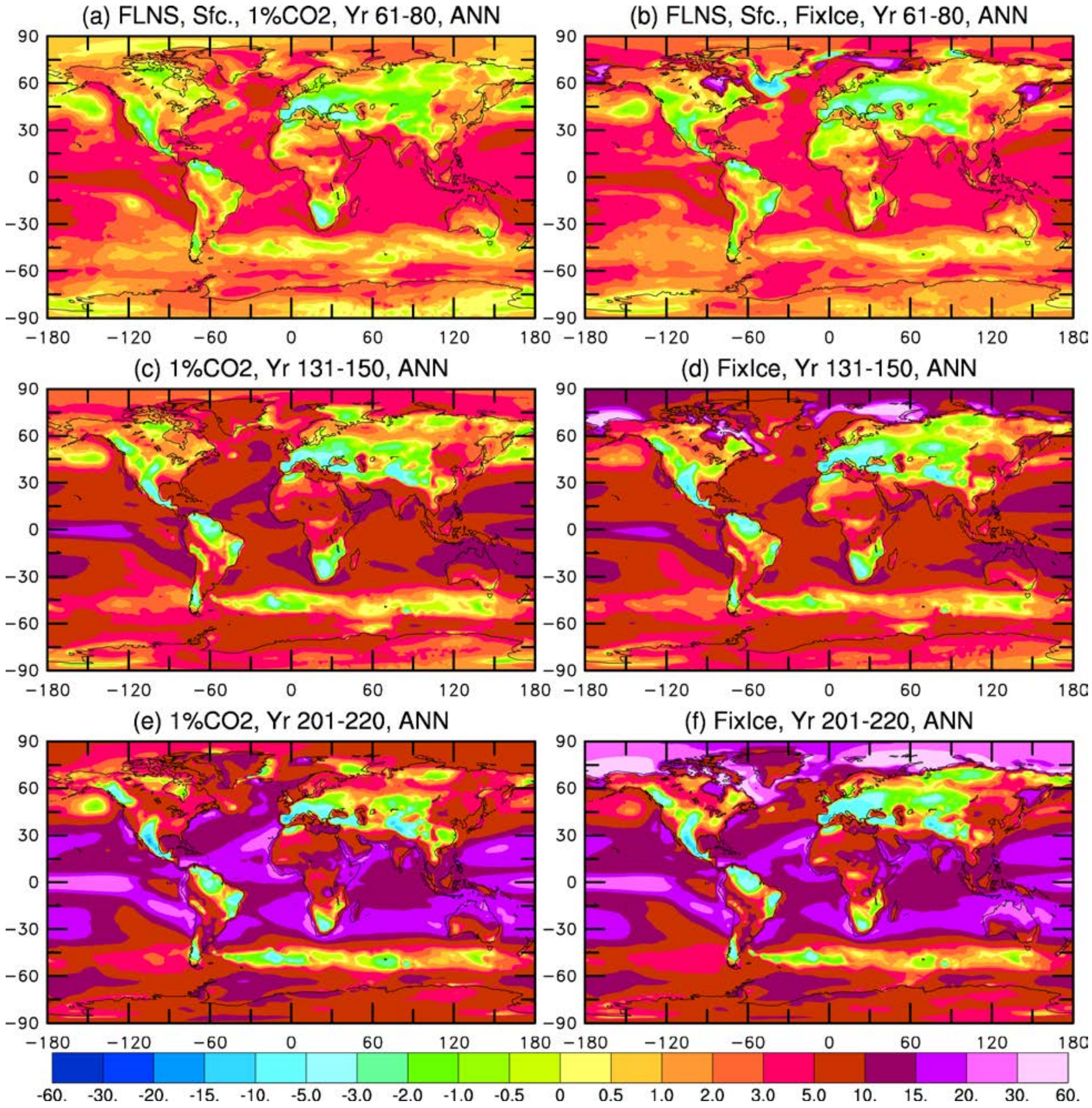

Figure 17. Changes (relative to control climatology) in annual surface net longwave (LW) radiative flux (W/m^2, positive downward) around the 1st (top row), 2nd (middle row) and 3rd (bottom row) doubling of atmospheric CO2 from the standard 1%CO2 (left) and Fixed_Ice (right) runs.

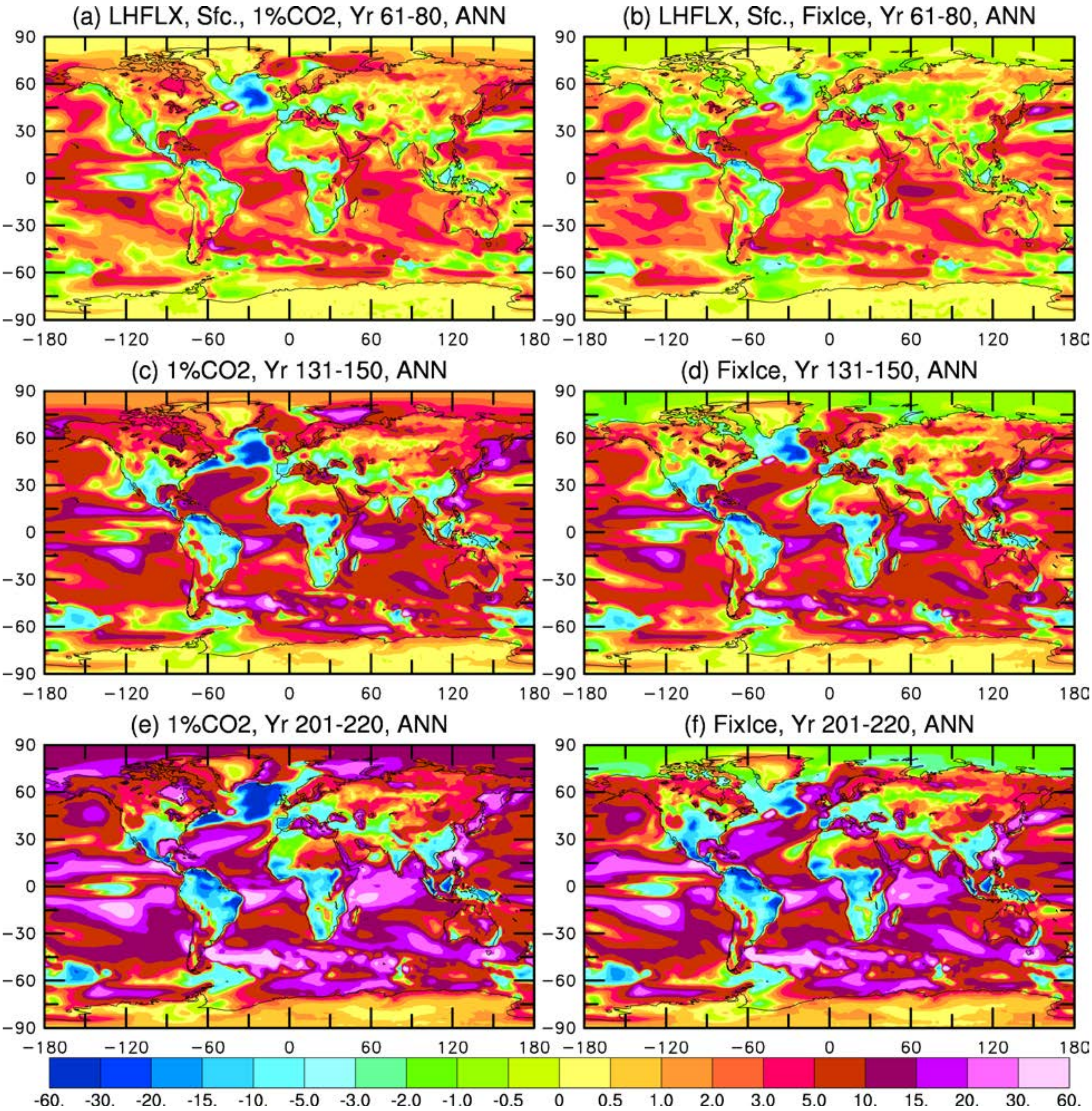

Figure 18. Changes (relative to control climatology) in annual surface latent heat flux (W/m^2, positive upward) around the 1st (top row), 2nd (middle row) and 3rd (bottom row) doubling of atmospheric CO2 from the standard 1%CO2 (left) and Fixed_Ice (right) runs.

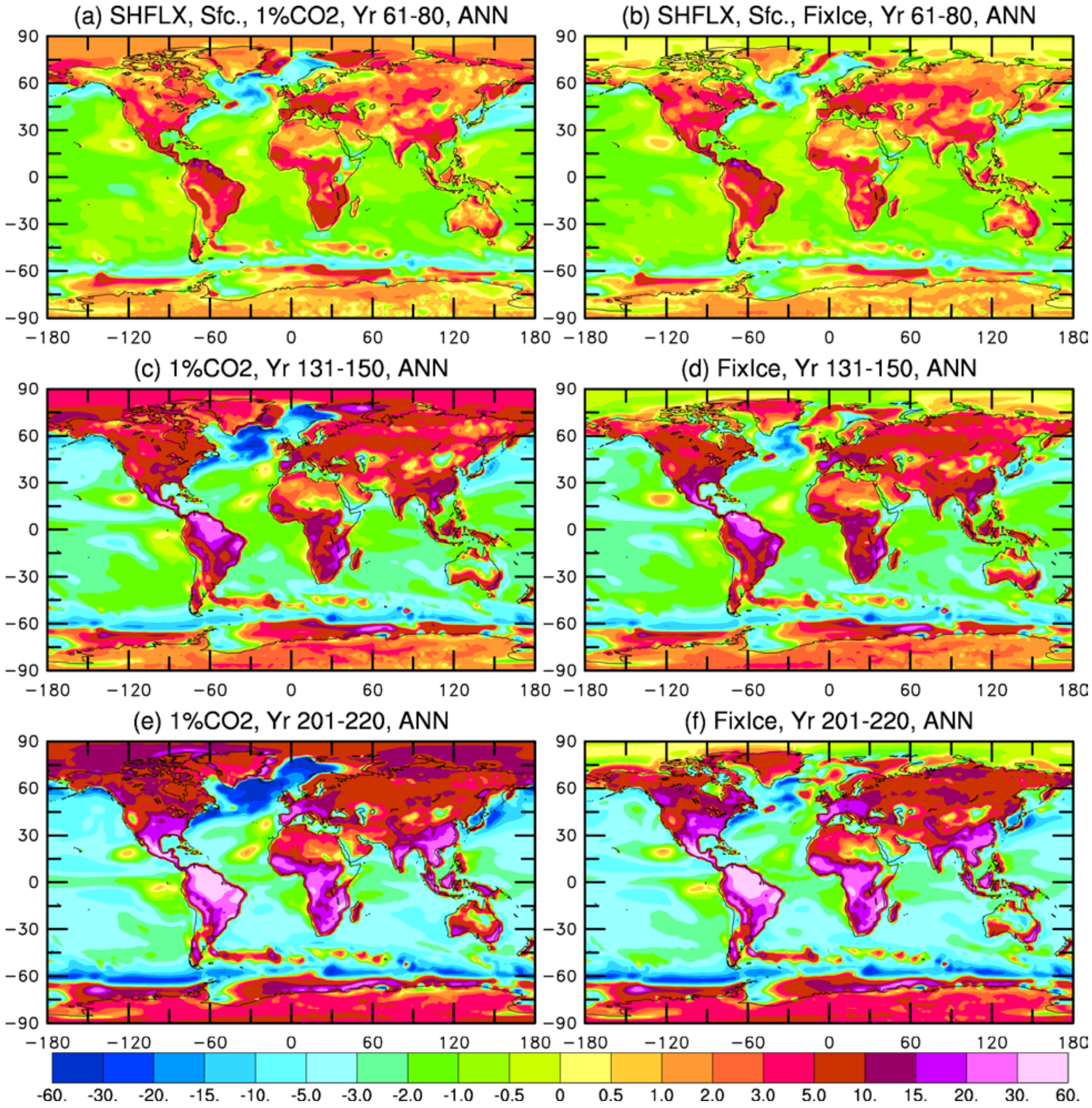

Figure 19. Changes (relative to control climatology) in annual surface sensible heat flux (W/m^2, positive upward) around the 1st (top row), 2nd (middle row) and 3rd (bottom row) doubling of atmospheric CO2 from the standard 1%CO2 (left) and Fixed_Ice (right) runs.
